# Depression in the nursing home: a cluster-randomized stepped-wedge study to probe the effectiveness of a novel case management approach to improve treatment (the DAVOS project)

**DOI:** 10.1186/s13063-019-3534-x

**Published:** 2019-07-11

**Authors:** Valentina A. Tesky, Arthur Schall, Ulrike Schulze, Ulrich Stangier, Frank Oswald, Monika Knopf, Jochem König, Maria Blettner, Elisabeth Arens, Johannes Pantel

**Affiliations:** 1 Department of Geriatric Medicine, Institute of General Practice, Goethe University Frankfurt, Theodor-Stern-Kai 7, 60590 Frankfurt am Main, Germany; 2 Hessian Institute of Nursing Research (HessIP), Frankfurt University of Applied Sciences (Frankfurt UAS), Nibelungenplatz 3, Frankfurt am Main, 60318 Germany; 3 Frankfurt Forum for Interdisciplinary Ageing Research (FFIA), Goethe University Frankfurt, Theodor-W.-Adorno-Platz 6, Frankfurt am Main, 60323 Germany; 4 Institute of Psychology, Department of Clinical Psychology and Psychotherapy, Goethe University Frankfurt, Varrentrappstraße 40-42, Frankfurt am Main, 60486 Germany; 5 Interdisciplinary Ageing Research, Faculty of Educational Sciences, Goethe University Frankfurt, Theodor-W.-Adorno-Platz 6, Frankfurt am Main, 60323 Germany; 6 Institute of Medical Biostatistics, Epidemiology and Informatics (IMBEI), Johannes Gutenberg University of Mainz, Langenbeckstraße 1, Mainz, 55131 Germany

**Keywords:** Late-life depression, Nursing home, Cluster-randomized intervention study, Case management, Psychotherapeutic treatment, Stepped-wedge design

## Abstract

**Background:**

Depression is the second most common psychiatric illness in old people. Up to 30% of nursing home residents have minor or major depression. Although depressive disorders in old age can be improved and even cured with adequate therapy, they often go unnoticed in nursing home residents and remain untreated. This highlights a striking deficit in health care and might result not only in lower quality of life among those concerned but also in poor physical functioning, premature mortality, and increased hospitalization rates.

**Methods:**

The aims of the interdisciplinary research project DAVOS are to implement an innovative and stepped structural case management program to improve depression treatment for nursing home residents by a modularized intervention and to assess it in terms of its effectiveness. Intervention modules are in line with recommendations given by the German national treatment guidelines for depression (S3 guidelines). Ten nursing homes in Frankfurt, Germany, will participate in the project, which aims to recruit a study population of 380. The recruitment will continue throughout the trial (open cohort). Persons (>60 years) who live in a nursing home, have no medical diagnosis of dementia, and can provide their informed consent to participate are eligible for inclusion in the study. Residents with a clinical diagnosis of dementia, alcohol or substance-related disorders, or other serious psychiatric illnesses will be excluded. DAVOS is a controlled cluster-randomized study that employs a stepped-wedge design.

**Discussion:**

Our main hypothesis is that the implementation of the intervention will lead to a decline in the prevalence of depression and a reduction in depression symptoms among the home residents. In addition, we expect the intervention to have a positive impact on secondary outcomes such as level of functioning, quality of life, and social participation. The project’s results can make an important contribution toward improving the health care of nursing home residents who have late-life depression.

**Trial registration:**

DRKS, DRKS00015686, Oct. 10, 2018.

## Background

Depression is the second most common psychiatric illness in old and very old people. Depending on the study, estimates of the prevalence of late-life depression (≥75 years of age) vary from 4.6% to 9.3% (pooled prevalence: 7.2%) for major depression and 4.5% to 37.4% (pooled prevalence: 17.1%) for milder forms of depressive illnesses (minor depression and persistent depressive disorder/dysthymia) [1]. At 6.8%, the incidence of clinically relevant depression symptoms in persons over 70 years of age is twice as high as the incidence of dementia [2, 3]. Furthermore, the varying presence of so-called subsyndromal symptomatic depression is common in later life and is a risk factor for the development of clinical depression. Further risk factors for late-life depression include frailty, multimorbidity, polypharmacy, and critical life events associated with old age, such as the loss of independence or a spouse [4]. Depression occurs significantly more often among old adults whose physical capacity is steadily declining and who are increasingly unable to participate in activities of daily living. Consequently, the prevalence of depression in nursing homes is almost twice as high as in late life in general: A field study conducted by Kramer et al. (2009) showed that around 30% of nursing home residents have acute depression (major depression: 14.4%; minor depression: 14.4%) [5]. Although depressive illnesses in old age can easily be treated, they often remain unnoticed and are not dealt with in nursing home residents. As a result of the structural peculiarities of the German health-care system, major depression is diagnosed in a mere 42.9% of nursing home residents who have the disease and only half of them receive an appropriate therapy [5]. This highlights a striking deficit in health care and results not only in lower quality of life among those concerned but also in poor physical functioning, premature mortality, and increased hospitalization [6–8]. Furthermore, the existence of chronic untreated depression has been shown to have a negative influence on the course of numerous somatic diseases as well as the risk of polypharmacy [9].

The aims of the project, known as “Depression in the nursing home: Using a stepped collaborative care model to improve treatment” (DAVOS: *Depression im Altenpflegeheim: Verbesserung der Behandlung durch ein gestuftes kollaboratives Versorgungsmodell*), are to implement an innovative and stepped structural case management program to improve depression treatment for nursing home residents by a modularized intervention and to assess it in terms of its effectiveness.

The main hypothesis of this research project is that the implementation of the intervention program will lead to a decline in the prevalence of depression and a reduction in the symptoms of depression (depression severity) among the residents of the participating nursing homes. Furthermore, it is expected to have a positive impact on secondary parameters that are influenced by depressive illnesses, such as level of functioning, quality of life, and social participation.

## Methods/Design

**Fig. 1:**
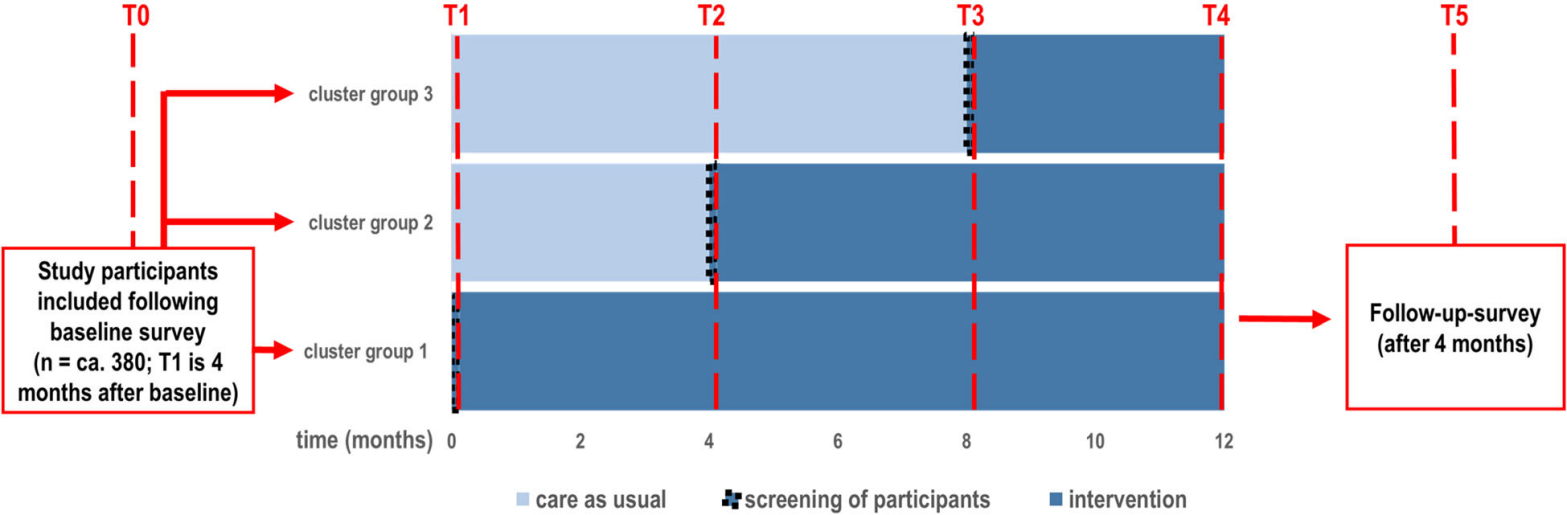
Study design

The DAVOS study has been approved by the ethics committee of the Goethe University of Frankfurt am Main, Germany (reference 129/18 and conforms to the Declaration of Helsinki, Version Fortaleza 2012). Trial registration is DRKS00015686 (www.drks.de). DAVOS is a controlled cluster-randomized trial that uses the stepped-wedge design. This type of waiting control group design employs repeated assessments, whereby each of the clusters (nursing homes) of the mentioned cluster group begins in the control phase. Each cluster group in DAVOS includes residents from three or four different nursing homes. After a certain delay, the intervention is gradually introduced in each cluster group (Fig. 1). During the waiting control phases, patients receive “usual care”. The point at which clusters pass from the control to the intervention phase is fixed. As soon as a new cluster group passes into the intervention phase, relevant data are collected from all clusters. In this way, data are gathered throughout the duration of the intervention (T1–T4) irrespective of whether the intervention has already been introduced. The T4 assessment can be classified as post-data collection. Furthermore, baseline (T0) and follow-up (T5) assessments are conducted 4 months before and 4 months after the intervention, respectively. Thus, depending on the time point of recruitment and inclusion in the study, residents will be repeatedly measured up to five times. Overall, 10 nursing homes (about 1250 care places) are included and randomly assigned to three cluster groups. We aim to recruit a study population of 380. As a result of newly admitted nursing home residents, the recruitment process will be continuous (open cohort), enabling the size of the study population included at baseline to be kept more or less constant throughout the study. In this way, the dropouts that are a natural part of the process, perhaps due to severe illness or death, can be compensated for and the number of assessments in each survey period can kept almost constant.

The stepped-wedge design allows comparisons to be made within and between the clusters receiving and not receiving the intervention. Furthermore, insights can be obtained into whether, and from what point, the complex intervention is effective. (The separate modules of the intervention have already been demonstrated to be effective in previous randomized studies.)

### Randomization

The nursing homes are the units of randomization (unrestricted randomization). We randomly assigned the 10 nursing homes (cluster) to the three cluster groups with computer-generated random numbers. We used online software to generate randomization by using a list randomizer (http://www.random.org). Finally, each cluster group includes residents from three or four different nursing homes. There are certain time points at which the cluster groups pass from the control to the intervention phase. All nursing homes and the included residents are informed about these time points.

### Setting and study population

**Fig. 2:**
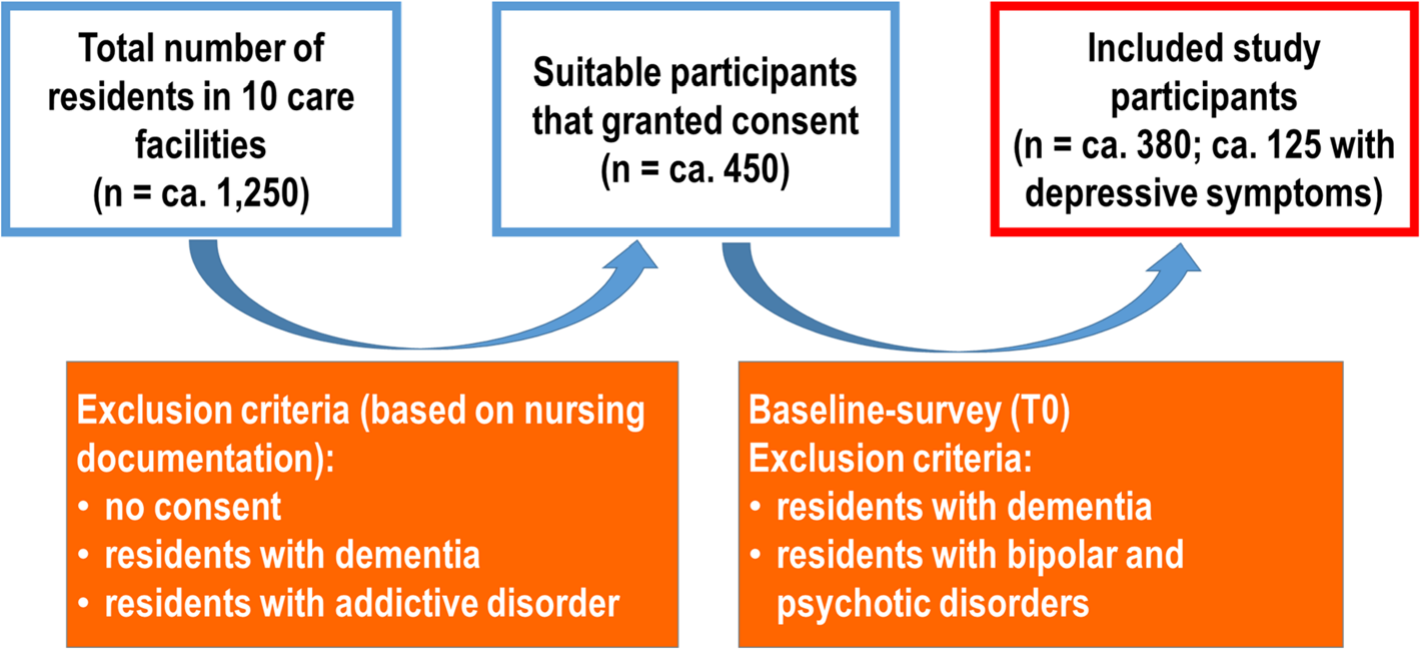
Participant recruitment process. Study participants with depression symptoms/depressive disorders (n = ⁓125) and without depression symptoms/depressive disorders (n = ⁓255)

The recruitment of participants and implementation of the intervention will take place at 10 outpatient nursing home facilities with a total of more than 1250 care places. This will be possible as a result of cooperation with the two social services organizations (German: *Frankfurter Verband für Alten- und Behindertenhilfe e.V.* and *Agaplesion Markus Diakonie gGmbH*). Based on nursing home records (e.g., on known diagnoses and health status), the nursing home residents who, in principle, are suitable for participation in the study will be approached by the previously appointed case managers. At this point, residents who have a clinical diagnosis of dementia (about 50%), are unable to provide consent, or have a known alcohol or substance-related disorder will be excluded from participation. It can be assumed from previous research that about one third of the overall population (about 36%) will be eligible for participation in the study and will provide their consent (Fig. 2). The data of residents who agree to participate in the study will be collected at baseline and checked against the inclusion and exclusion criteria.

Residents over 60 years of age and without obvious signs of dementia, an addictive disorder, or another severe mental illness will be included in the study. Inclusion criteria for participation in the interventional modules as described below are the presence of a subsyndromal depressive disorder or clinical depression. It makes sense to include subsyndromal symptomatic depression because it is common in old age and defines an important target population for the use of secondary preventive measures. In any case, diagnosis of subsyndromal or clinically manifest depression is based on ICD 10 (10th revision of the International Statistical Classification of Diseases and Related Health Problems) criteria following the judgment of a psychotherapist who is licensed by a state board (in Germany: *Approbierter Psychotherapeut*). The establishment of a proper clinical diagnosis is already part of the intervention as described below.

### Intervention

**Fig. 3:**
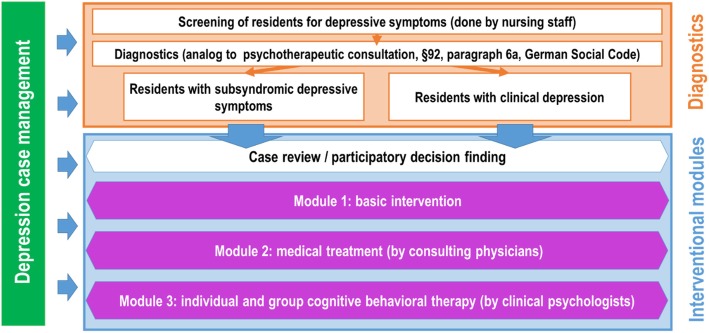
Presentation of the case management program and the interventional modules

The case management program and the interventional modules are shown in Fig. 3. The depression case managers play an important integrative role in the intervention, as they are at the interface between residents, nurses, physicians, and psychotherapists. The case managers are nurses who have been selected by the management of the respective nursing homes and who have been trained for this role before the intervention begins. In every participating nursing home, two depression case managers are selected. Altogether, 20 depression case managers are trained during the study. The case manager’s tasks include the identification of suitable study participants, prompt presentation during the psychotherapeutic consultation hour (see below) in the case of positive screening of depression, and coordination of the treatment modules for the participants.

The training for case managers will include the following four elements: (1) communication of basic medical-psychological information on late-life depression, (2) use of the screening instrument, (3) information on how to deal with residents with depression, and (4) the organization of project-related requirements. Case managers will be supervised throughout the study.

The intervention is initiated by a screening applied to the participating residents using a modified version of the Depression Monitoring List (DeMoL) with integrated Patient Health Questionnaire (PHQ-D) assessment [10]. The screening is performed by the depression case manager or by other members of the nursing staff under the supervision of the case manager. In case of positive screening, the participant is referred to a psychotherapeutic consultation hour (in German: *Psychotherapeutische Sprechstunde*) in accordance with §92, paragraph 6a, German Social Code (in German: *Sozialgesetzbuch*), during which a board-licensed psychological psychotherapist will provide a diagnostic assessment (according to ICD 10 criteria). As part of DAVOS, the psychotherapeutic consultation hour will be implemented as an “in house” service in the nursing home; this is an innovative approach compared with the usual practice in the German health-care system. The assessment in the psychotherapeutic consultation hour will conclude with recommendations for several interventions that are elaborated in accordance with the German S3 guideline and the National Disease Management Guideline on Unipolar Depression [6] and are part of three interventional modules. Ranging from “watchful waiting”, participation in basic intervention (module 1), and a recommendation for psychotherapy (module 3) to the involvement of the general practitioner or a specialist physician (e.g., psychiatrist) (module 2), the measures will cover a wide spectrum of possible interventions:

Module 1 (basic intervention) consists of participation in group sessions that are offered to all participants with or without any symptoms of depression (including persons with subsyndromal depressive disorders). Key components of this module are supportive and psychoeducative approaches (on the subjects of “successful ageing”, “mindfulness”, etc.). By preparing a weekly plan, for example, the residents are to be animated to participate in measures involving physical activity as well as other social and leisure pursuits. The case managers initially assist to carry out this module but later take on full responsibility for it. The aims are to establish this basic intervention as part of the daily nursing routine and to encourage everyday companions and other nurses to use it after the intervention is over. The case managers are thus to adopt the role of multipliers.

Module 2 contains aspects of treatment that require the therapeutic involvement of the general practitioner in charge of the resident or a specialist physician or both (such as exclusion or treatment of somatic causes of depression, drug therapy/antidepressants, interactions with other drugs, polypharmacy, and hospital admissions). The role of the case manager here is to prompt and coordinate appointments with the doctors in charge following recommendations derived from the psychotherapeutic consultation hour.

Module 3 covers participation in psychotherapeutic groups and, where applicable, individual psychotherapy conducted by psychologists. The employed interventions include elements of cognitive behavioral therapy (CBT) (e.g., planning pleasant daily activities, problem solving, mindfulness-based meditation, and cognitive restructuring [6, 11–15]). If required, individual psychotherapy sessions will be provided to residents with major depression, dysthymia, and adjustment disorders. Psychotherapy will partly be delivered by psychologists in clinical training from the outpatient clinic of the Department of Clinical Psychology and Psychotherapy at Goethe University Frankfurt. The psychologists will receive additional training in CBT for late-life depression [16] and mindfulness-based cognitive therapy [14, 15].

The psychologists’ tasks are (1) to participate in regular meetings at the nursing home facilities with case managers to ensure exchange of information about cases and treatments, (2) to conduct psychotherapeutic consultations in order to motivate the patient for psychotherapy and select a suitable setting, (3) and to conduct either individual or group CBT, including elements of mindfulness-based meditation [6, 11–15]). After two months, individuals who meet the inclusion criteria for the intervention but do not wish to participate will be contacted again and informed about the provided treatment.

### Data collection

Questionnaires and psychometric instruments that have been validated in clinical and gerontology research will be used to collect data face to face. For this purpose, instruments have been selected that are well established and time-efficient but that simultaneously cover a wide range of outcome-relevant variables. Data collection will be mainly quantitative but also supplemented by qualitative methods (e.g., interviews with case managers and use of focus groups). These qualitative data will be analyzed by the sequential analysis based on grounded theory and on the research of Rosenthal [17] and grounded theory [18].

Subsequently, paper-and-pencil data will be digitalized, checked, and subjected to missing values analysis. Questionnaires with more than 30% of values missing will not be included in the subsequent analysis. An estimate of isolated missing values will be made by using, for example, the full information maximum likelihood algorithm.

The raters responsible for data collection are part of the study team but are not involved in the intervention (e.g., psychotherapy). They will receive intensive training in using the deployed instruments (test methods and questionnaires). The exclusive use of standardized instruments will ensure that the influence of individual raters is negligible and that the data are valid. During training, the trustworthiness of the data collection process will also be controlled by calculating inter-rater reliability.

### Outcomes

The prevalence of depressive disorders and the severity of depression symptoms (or any change in them) among nursing home residents are the primary outcomes of DAVOS. Secondary outcomes are quality of life, functional status (instrumental activities of daily living), social participation, and the type, frequency, and duration of any hospitalization during the observation period. In addition to data on primary and secondary outcomes (T0 to T5), relevant personal and sociodemographic data will be collected at baseline (including family background, socioeconomic status, educational level, subjective health status [19], personality characteristics [20], cognitive status [21], current medication, and somatic comorbidities). To minimize the stress of data assessment, nursing home documentation can serve as an additional data source. Some of the variables, such as health status and cognitive status, will be measured repeatedly at the T5 follow-up assessment. All instruments are well introduced and validated and have frequently been used in previous research. An overview of the instruments and associated validation references is presented in Table 1.

**Table 1:** Target variables and survey instruments of DAVOS

Baseline and follow-up assessments (T0 and T5) in addition to outcomes	
Variables/instruments	
Sociodemographic variables: age, gender, family background, and further relevant sociodemographic data (*only T0*)	
Subjective health status: 12-Item Short-Form Health Survey (SF-12_v2)	
Personality characteristics: Big-Five Inventory Short Version (BFI-10)	
Dementia screening: Mini-Mental State Examination (MMSE)	
Primary outcomes (T0-T5)	
Variables/instruments	
Prevalence of depression, dysthymia, adjustment disorders: Structured Clinical Interview for the fourth edition of the *Diagnostic and Statistical Manual of Mental Disorders* (SCID-I)	
Severity of depression symptoms: Geriatric Depression Scale (GDS)	
Secondary outcomes (T0-T5)	
Variables/instruments	
Frequency and duration of hospitalizations: Nursing home records	
Quality of life: World Health Organization Quality of Life, short form (WHOQoL Old)	
Quality of life (Attitude toward own aging): Philadelphia Geriatric Center Morale Scale (PGCMS)	
Functional activity level: Late Life Function and Disability Instrument, short form (SF-LLFDI)	
Social participation: Social and Emotional Loneliness Scale - short form	

*Abbreviations*: *DAVOS* Depression in the nursing home: Using a stepped collaborative care model to improve treatment (*Depression im Altenpflegeheim: Verbesserung der Behandlung durch ein gestuftes kollaboratives Versorgungsmodell*), *T* time point

The primary outcomes (prevalence of depressive disorders and severity of depression symptoms) will be assessed by using the structured clinical interview for the fourth edition of the *Diagnostic and Statistical Manual of Mental Disorders* (SCID-I) [22] and the Geriatric Depression Scale (GDS) [23]. SCID-I is a commonly used means of recording and diagnosing selected mental syndromes and disorders. The 15 items on the GDS are part of the geriatric assessment for the evaluation of depressive disorders in old age.

Baseline assessments are conducted simultaneously in all of the clusters to determine prevalence rates of depression at differing levels of severity. We expect a significant decrease of prevalence rates of depression as well as severity of depression symptoms in the clusters that receive interventions, as compared with clusters receiving care as usual (stepped-wedge approach). The screenings conducted by the case managers that are part of the intervention are independent of the outcome assessments and therefore cannot result in an increase in prevalence.

Established instruments and scales which are developed especially for old adults will be used to measure secondary outcomes such as quality of life, functional competence, and social participation. Examples of these are the Philadelphia Geriatric Center Morale Scale (PGCMS) [24], the World Health Organization (WHO) Quality of Life (short form) (WHOQOL-OLD) [25, 26], a questionnaire for the assessment of aspects of quality of life in later life, the short form of the Late Life Function and Disability Instrument (SF-LLFDI) [27], and the Social and Emotional Loneliness Scale (short form) [28]. Information on the frequency and duration of hospitalization will be taken from nursing home records.

### Data analysis and data quality assurance

The evaluation will be conducted by using a stepped-wedge design with open cohorts [29]. That means that not only at the beginning but also during the course of the trial, new participants will be recruited into each of the 10 clusters. Each new recruit will generally undergo all of the data assessments (periods) from inclusion in the trial until either he or she leaves the nursing home or the project ends. The percentage (point prevalence) of participants with depression will be assessed at the end of each period; some of the trial participants will be assessed for the first time and others not. If the new care model is effective, the prevalence of depression is expected to decline significantly. The use of a stepped-wedge design ensures that results do not reflect a general period effect, meaning that they do not result from a change in prevalence in all the clusters, independently of whether a cluster is in the control or intervention phase.

A hierarchical generalized linear model with a logit link function will be used for the analysis and will account for not only fixed treatment and period effects but also random effects on both cluster and patient levels. In order to analyze the second primary endpoint (depression severity), we will fit a hierarchical logistic regression model for ordinal target variables (cumulative odds model). Details will be laid down in a statistical analysis plan. The statistical models mentioned above are suitable for use with correlated categorical data for the incomplete courses that the planned design leads us to expect. Bias can be avoided or at least limited through the use of randomization and the standardization of data collection methods.

Based on a sample size of 38 participants to be assessed in each cluster (nursing home) in each period, cluster groups of three, four, and three (10 nursing homes), a type 1 error probability of 0.05 in a two-tailed test, a prevalence (including subsyndromal symptomatic depression) of 33% under control conditions [30] and 22% under intervention conditions, and assuming a between-cluster standard deviation of prevalences of 4%, and assuming independence between repeated observations on the same patients within clusters across periods, the power of the study will be 0.80. We assumed a time constant random cluster effect, which induces a correlation structure with an intraclass coefficient of 0.0072 under the null hypothesis and of 0.0092 under alternative hypothesis within intervention periods, both within periods and between periods. Note that this assumption also implies a perfect autocorrelation of random cluster effects across time. For power calculation, we used the R package swCRTdesign [31]. In fact, patient-level random effects will introduce dependencies within clusters across periods. This induces a reduction in effective sample size for repeated observation under the same treatment and increased effective sample size for repeated observation under different treatments. We expect that the latter effect will dominate because the majority of patients will be observed under both treatment conditions. At the planning stage of the project, we did not perform further sensitivity analyses to account for variability in cluster size, for attrition, and for more realistic correlation structures. For attrition, we expect a limited impact because new patients will be recruited while the study is ongoing to account for eventual dropout and patients dying.

## Discussion

Collaborative and multiprofessional treatment concepts are an effective and efficient means of providing appropriate health care for old and very old persons and have recently been recommended for the treatment of depression in nursing home residents [32, 33]. Nevertheless, there are a few health-care programs available that address the problem of late-life depression in Germany [11]. The results of an RCT on the effectiveness of the Dutch program “Act in Case of Depression” (AiD) represent one of the few records of a program for the treatment of depression in non-demented residents of nursing homes. Previous results show that the implementation of AiD in Dutch nursing homes led to a significant decline in the prevalence of depression and an improvement in quality of life among residents [34–36]. Based on the experiences gathered in the AiD program, DAVOS will develop and implement a comprehensive case management program for institutionalized long-term care settings in Germany and conduct a controlled cluster-randomized trial to evaluate it by an interdisciplinary group of experts.

The program addresses important reasons for the health-care deficits related to the adequate treatment of depression in German nursing homes. These are, first, that depressive illnesses (including the subsyndromal symptomatic depression that is more common in later life) are often recognized too late or not at all. Second, previously described structural peculiarities also play a role, as they generally complicate the provision of appropriate medical treatment for nursing home residents (e.g., interface problems such as insufficient coordination and cooperation between nurses and doctors) [37]. In Germany, in addition to structural obstacles that are typical in a decentrally organized and fragmented health-care system, appropriate treatment of depression in nursing home residents is complicated by certain legal conditions and guidelines. One of these obstacles is the high threshold for licensed psychotherapists in providing and billing psychotherapeutic treatment in nursing homes. In this respect, the results and practical experiences gained during the DAVOS project can be expected to give indications of how, in the future, needs-oriented health care can be provided to nursing home residents with depression.

## Trial status

At the time the manuscript was submitted, the first participants had been recruited into the study. Recruitment began December 20, 2018. The study is expected to be completed in March 2021. Trial registration: DRKS, DRKS00015686 (10.10.2018). Protocol version 1.0 12.02.2019 VT.

## Data Availability

Not applicable.

## Abbreviations

AiD: Act in Case of Depression
CBT: Cognitive behavioral therapy
DAVOS: *Depression im Altenpflegeheim: Verbesserung der Behandlung durch ein gestuftes kollaboratives Versorgungsmodell* (Depression in the nursing home: Using a stepped collaborative care model to improve treatment)
GDS: Geriatric Depression Scale
ICD 10: 10th revision of the International Statistical Classification of Diseases and Related Health Problems
n: Sample size
SCID-1: Structured clinical interview for the fourth edition of the *Diagnostic and Statistical Manual of Mental Disorders*
T: Time point
